# Tissue-Specific Signatures in the Transcriptional Response to *Anaplasma phagocytophilum* Infection of *Ixodes scapularis* and *Ixodes ricinus* Tick Cell Lines

**DOI:** 10.3389/fcimb.2016.00020

**Published:** 2016-02-10

**Authors:** Pilar Alberdi, Karen L. Mansfield, Raúl Manzano-Román, Charlotte Cook, Nieves Ayllón, Margarita Villar, Nicholas Johnson, Anthony R. Fooks, José de la Fuente

**Affiliations:** ^1^SaBio, Instituto de Investigación en Recursos Cinegéticos IREC-Consejo Superior de Investigaciones Científicas- Universidad de Castilla–La Mancha-Junta de Comunidades de Castilla–La ManchaCiudad Real, Spain; ^2^Animal and Plant Health AgencyNew Haw, Surrey, UK; ^3^Parasitología Animal, Instituto de Recursos Naturales y Agrobiología de Salamanca (IRNASA, Consejo Superior de Investigaciones Científicas)Salamanca, Spain; ^4^Department of Clinical Infection, Microbiology and Immunology, University of LiverpoolLiverpool, UK; ^5^Department of Veterinary Pathobiology, Center for Veterinary Health Sciences, Oklahoma State UniversityStillwater, OK, USA

**Keywords:** transcriptomics, tick, rickettsia, anaplasma, apoptosis, tick cell line

## Abstract

*Anaplasma phagocytophilum* are transmitted by *Ixodes* spp. ticks and have become one of the most common and relevant tick-borne pathogens due to their impact on human and animal health. Recent results have increased our understanding of the molecular interactions between *Ixodes scapularis* and *A. phagocytophilum* through the demonstration of tissue-specific molecular pathways that ensure pathogen infection, development and transmission by ticks. However, little is known about the *Ixodes ricinus* genes and proteins involved in the response to *A. phagocytophilum* infection. The tick species *I. scapularis* and *I. ricinus* are evolutionarily closely related and therefore similar responses are expected in *A. phagocytophilum*-infected cells. However, differences may exist between *I. scapularis* ISE6 and *I. ricinus* IRE/CTVM20 tick cells associated with tissue-specific signatures of these cell lines. To address this hypothesis, the transcriptional response to *A. phagocytophilum* infection was characterized by RNA sequencing and compared between *I. scapularis* ISE6 and *I. ricinus* IRE/CTVM20 tick cell lines. The transcriptional response to infection of *I. scapularis* ISE6 cells resembled that of tick hemocytes while the response in *I. ricinus* IRE/CTVM20 cells was more closely related to that reported previously in infected tick midguts. The inhibition of cell apoptosis by *A. phagocytophilum* appears to be a key adaptation mechanism to facilitate infection of both vertebrate and tick cells and was used to investigate further the tissue-specific response of tick cell lines to pathogen infection. The results supported a role for the intrinsic pathway in the inhibition of cell apoptosis by *A. phagocytophilum* infection of *I. scapularis* ISE6 cells. In contrast, the results in *I. ricinus* IRE/CTVM20 cells were similar to those obtained in tick midguts and suggested a role for the JAK/STAT pathway in the inhibition of apoptosis in tick cells infected with *A. phagocytophilum*. Nevertheless, tick cell lines were derived from embryonated eggs and may contain various cell populations with different morphology and behavior that could affect transcriptional response to infection. These results suggested tissue-specific signatures in *I. scapularis* ISE6 and *I. ricinus* IRE/CTVM20 tick cell line response to *A. phagocytophilum* infection that support their use as models for the study of tick-pathogen interactions.

## Introduction

*Anaplasma phagocytophilum* (Rickettsiales: Anaplasmataceae) is the causative agent of human granulocytic anaplasmosis (HGA), equine and canine granulocytic anaplasmosis, and tick-borne fever of ruminants (TBF; Severo et al., 2015). *A. phagocytophilum* are transmitted by *Ixodes scapularis* and *I. pacificus* in the United States and by *I. ricinus* in Europe becoming one of the most common and relevant tick-borne pathogens in these regions due to their impact on human and animal health (Goodman, 2005; Stuen, 2007; Severo et al., 2015). Furthermore, the wide host range of *A. phagocytophilum* (Estrada-Peña et al., 2015) and the extensive distribution and expansion of tick vector populations (Estrada-Peña et al., 2014) will likely make this tick-borne pathogen a growing concern for human and animal health worldwide.

The *I. scapularis* genome is the only tick genome sequenced and assembled (Geraci et al., 2007) and constitutes a valuable resource for the study of tick biology and tick-pathogen interactions with particular interest for closely related species such as *I. ricinus* (Genomic Resources Development Consortium et al., 2014). Recent results have increased our understanding of *I. scapularis*–*A. phagocytophilum* interactions through the demonstration of tissue-specific molecular pathways that ensure *A. phagocytophilum* infection, development and transmission by ticks (Ayllón et al., 2013, 2015a; Villar et al., 2015a,b). However, little is known about the genes and proteins of *I. ricinus* involved in the response to *A. phagocytophilum* infection (Alberdi et al., 2015; Ayllón et al., 2015b).

Recently, Alberdi et al. (2015) demonstrated that different geographic isolates of *A. phagocytophilum* inhibit apoptosis in both *I. scapularis* ISE6 and *I. ricinus* IRE/CTVM20 tick cells, supporting that pathogen infection inhibits apoptotic pathways to facilitate infection in different tick vector species. However, infection with *A. phagocytophilum* inhibited the intrinsic apoptosis pathway at different levels in *I. scapularis* and *I. ricinus* cells, suggesting that differences may exist between tick species in response to infection (Alberdi et al., 2015). Alternatively, as has been shown in *I. scapularis* midguts and salivary glands (Ayllón et al., 2015a) and ISE6 cells (Villar et al., 2015a), tick cell lines may reflect tissue-specific differences in response to *A. phagocytophilum* infection. The *I. scapularis* ISE6 and *I. ricinus* IRE/CTVM20 tick cell lines were derived from embryonated eggs and contain cells with different morphology and behavior (Munderloh et al., 1994; Bell-Sakyi et al., 2007).

The *I. scapularis* and *I. ricinus* cells infected with *A. phagocytophilum* may show different response to infection due to (a) differences between tick species or (b) differences between *I. scapularis* ISE6 and *I. ricinus* IRE/CTVM20 tick cells derived from tissue-specific signatures of these tick cell lines derived from embryonated eggs. However, due to the close evolutionary relationship between *I. scapularis* and *I. ricinus* (Pedra et al., 2010; Dyachenko et al., 2013; Schwarz et al., 2013; Genomic Resources Development Consortium et al., 2014), our hypothesis is that differences between *I. scapularis* and *I. ricinus* tick cells in response to *A. phagocytophilum* are the result of tissue-specific signatures of these tick cells. To address this hypothesis, the transcriptional response to *A. phagocytophilum* infection was compared between *I. scapularis* ISE6 and *I. ricinus* IRE/CTVM20 tick cell lines. The results suggested tissue-specific signatures in tick cell line response to *A. phagocytophilum* infection that support their use as models for the study of tick-pathogen interactions.

## Materials and methods

### *I. scapularis* and *I. ricinus* tick cells and sample preparation

The *I. scapularis* embryo-derived tick cell line ISE6 (provided by Ulrike Munderloh, University of Minnesota, USA) was cultured in L-15B300 medium and used in these experiments as previously described (Villar et al., 2015a). The *I. ricinus* embryo-derived tick cell line IRE/CTVM20 (provided by Lesley Bell-Sakyi, Tick Cell Biobank, Pirbright Institute, UK) was maintained in a 1:1 mixture of L-15 (Leibovitz) medium and L-15B medium (Munderloh and Kurtti, 1989) as previously described (Bell-Sakyi et al., 2007). Tick cells were inoculated with *A. phagocytophilum* (human NY18 isolate) as previously described (Villar et al., 2015a). Mock infected (uninfected) and infected cells (two independent cultures with approximately 10^7^ cells each) were sampled at 7 days post-infection (dpi) [percent infected cells: ISE6, 71-77% (Ave ± SD, 74 ± 3); IRE/CTVM20, 58-62% (Ave ± SD, 60 ± 2)]. The percentage of *A. phagocytophilum* infected cells was calculated by examining at least 200 cells using a 100x oil immersion objective. The cells were centrifuged at 10,000 × g for 3 min, and cell pellets were frozen in liquid nitrogen until used for RNA extraction. Total RNA was extracted using TRIzol (Invitrogen, Carlsbad, CA, USA) following the manufacturer's recommendations. The RNA was further purified using the RNeasy Mini kit (Qiagen, Venlo, Netherlands). Ribosomal RNA (rRNA) was depleted using Terminator exonuclease (Epicentre, Madison, WI, USA) according to the manufacturer's instructions. Prior to sequencing, the RNA samples were quantified by spectrophotometry to confirm that each sample was at a suitable concentration (>10 ng/μl) for RNA sequencing (RNAseq). The RNA was then used for RNAseq and real-time RT-PCR.

### RNAseq and analysis

RNAseq was undertaken for both tick cell lines with duplicate RNA samples from uninfected and infected cells at each time point. For *I. scapularis* cell line ISE6, RNAseq was conducted as reported previously (Villar et al., 2015a). For *I. ricinus* cell line IRE/CTVM20, RNAseq was undertaken using a similar experimental approach. Briefly, RNA (200 ng) was reverse transcribed to generate double-stranded cDNA using the cDNA Synthesis System (Roche, Basel, Switzerland) and random hexamers. Illumina sequencing libraries were prepared using the Nextera XT system (Illumina, San Diego, CA, USA) and sequenced using an Illumina GA IIx instrument. Sequence analysis was undertaken using multiplexed paired-end samples. Pre-analysis sequence quality checking was performed using the FastQC programme (Babraham Institute, Babraham, Cambridgeshire, United Kingdom). The program BowTie2 (http://bowtie-bio.sourceforge.net/bowtie2/index.shtml) was used as an assembler to align sequenced reads with the reference *I. scapularis* genome sequence (assembly JCVI_ISG_i3_1.0; http://www.ncbi.nlm.nih.gov/nuccore/NZ_ABJB000000000). TopHat2 was then used to analyze the mapping results and identify splice junctions between exons. The Cufflinks program was used to provide an estimation of gene abundance and differential gene expression, allowing for splice variants and gaps due to the genome reference. Within Cufflinks, Cuffmerge was used to merge Cufflinks assemblies to provide normalization of biological replicates. Cuffquant was used to provide abundance estimation across normalized samples. The Cuffdiff algorithm was used to account for biological variability between samples and identify differentially expressed genes; this included non-statistical analysis (log2 fold-change) and statistical analysis (test for variance), in order to identify statistically significant fold-changes in gene expression (*p* ≤ 0.05; *q* ≤ 0.05). The TopHat-Cufflinks-Cuffmerge-Cuffquant-Cuffdiff pipeline was also used to analyze RNAseq data for *I. scapularis* cell line ISE6 (Villar et al., 2015a) and differentially expressed genes were selected for this study based on the same criteria used for the *I. ricinus* cell line IRE/CTVM20 (*p* ≤ 0.05; *q* ≤ 0.05). The RNAseq data for *I. scapularis* cell line ISE6 were deposited in NCBI's Gene Expression Omnibus and are accessible through GEO Series accession number GSE68881 (http://www.ncbi.nlm.nih.gov/geo/query/acc.cgi?acc=GSE68881; Villar et al., 2015a). For *I. ricinus* cell line IRE/CTVM20, the RNAseq data have been deposited in NCBI's Gene Expression Omnibus and are accessible through GEO Series accession number GSE76906 (http://www.ncbi.nlm.nih.gov/geo/query/acc.cgi?acc=GSE76906).

### Bioinformatics data analysis and gene ontology (GO) assignments

Functional annotations for each gene were obtained from Uniprot (www.uniprot.org) using GO annotations, Enzyme Commission (EC) number, and InterPro (www.ebi.ac.uk/interpro) using DAVID functional annotation tool (http://david.abcc.ncifcrf.gov/tools.jsp). Configuration for GO annotation included an *E*-value-Hit-filter of 1.0E-6, annotation cut off of 55, and GO weight of 5. For visualizing the GO annotations for molecular function (MF) and biological process (BP), the analysis tool of the Blast2GO software (version 2.6.6; www.blast2go.org) was used.

The InterPro motifs obtained using DAVID were used to evaluate the fold enrichment of differentially expressed genes. Functional annotation provides a Chart Report containing an annotation-term-focused view, which lists annotation terms and their associated genes under study. To avoid over counting duplicated genes, the Fisher Exact statistics was calculated based on corresponding DAVID gene IDs in which all redundancies in original IDs were removed. The results of Chart Report have to pass the thresholds (by default, Maximum Probability ≤ 0.1 and Minimum Count ≥ 2) in Chart Option section to ensure that only statistically significant ones are displayed. To evaluate the fold enrichment of differentially expressed genes, which corresponds to a set of genes highly associated with certain terms, the EASE Score Threshold (Maximum Probability) was used. The threshold of EASE Score, a modified Fisher Exact *p*-value, ranges from 0 to 1. Fisher Exact *p* = 0 represents perfect enrichment. We used Fisher Exact *p* ≤ 0.05 to consider enrichment in the annotation categories for differentially expressed genes. Finally, Panther (www.pantherdb.org) was used to calculate overrepresented and underrepresented GO categories. The fold enrichment (FE) of the genes observed in the uploaded list is divided by the expected number. If it is >1, then the category is overrepresented in the dataset. Conversely, the category is underrepresented if it is less than 1. For all BP and MF categories in upregulated and downregulated genes, values were compared between tick cell lines by Chi^2^ test (*p* = 0.01).

### Characterization of tick mRNA levels by real-time RT-PCR

The expression of selected differentially expressed genes was characterized using total RNA extracted from infected and uninfected ISE6 and IRE/CTVM20 tick cells. Real-time RT-PCR was performed on RNA samples using gene-specific oligonucleotide primers (Supplementary Table 1) and the iScript One-Step RT-PCR Kit with SYBR Green and the CFX96 Touch Real-Time PCR Detection System (Bio-Rad, Hercules, CA, USA). A dissociation curve was run at the end of the reaction to ensure that only one amplicon was formed and that the amplicons denatured consistently in the same temperature range for every sample. The mRNA levels were normalized against tick 16S rRNA and cyclophilin using the genNorm method (ddCT method as implemented by Bio-Rad iQ5 Standard Edition, Version 2.0) as previously described (Ayllón et al., 2013). Normalized Ct values were compared between infected and uninfected tick cells by Student's *t*-test with unequal variance (*P* = 0.05; *N* = 5 biological replicates).

## Results and discussion

### Transcriptional response to *A. phagocytophilum* infection in *I. scapularis* ISE6 and *I. ricinus* IRE/CTVM20 cells

The RNAseq and differential expression analysis in *A. phagocytophilum*-infected *I. scapularis* cell line ISE6 was reported previously (Villar et al., 2015a). A total of 37,990 transcripts were identified of which 174 corresponded to differentially expressed genes between infected and uninfected cells (*p* ≤ 0.05; *q* ≤ 0.05) and used for this study (Supplementary Table 2). Of them, 45 and 129 genes were upregulated and downregulated, respectively in infected cells when compared to uninfected cells (Supplementary Table 2).

The RNAseq in *A. phagocytophilum*-infected *I. ricinus* cell line IRE/CTVM20 resulted in 20,681 genes (Supplementary Tables 3, 4). Differential expression analysis identified 197 differentially expressed genes between infected and uninfected cells (*p* ≤ 0.05; *q* ≤ 0.05; Supplementary Table 5). Of them, 154 and 43 genes were upregulated and downregulated, respectively in infected cells when compared to uninfected cells (Supplementary Table 5).

The analysis of transcriptomics results in *I. scapularis* ISE6 and *I. ricinus* IRE/CTVM20 tick cells showed that despite similar numbers of differentially expressed genes in response to infection, the number of upregulated and downregulated genes varied between cell lines. Additionally, of the differentially regulated genes only 36 were identified in both cell lines, with differences in response to infection (Table 1). These results evidenced differences in the transcriptional response to *A. phagocytophilum* infection of *I. scapularis* ISE6 and *I. ricinus* IRE/CTVM20 cells.

**Table 1:** **Differentially expressed genes identified in both *I. ricinus* IRE/CTVM20 and *I. scapularis* ISE6 cell lines**.

**Protein description**	**Accession number**
**GENES UPREGULATED IN BOTH CELL LINES**	
Cell adhesion molecule, putative	ISCW001458
Organic cation/carnitine transporter, putative	ISCW008146
Voltage-gated ion channel, putative	ISCW023559
**GENES DOWNREGULATED IN BOTH CELL LINES**	
Neural cell adhesion molecule L1, putative	ISCW023403
ABC transporter, putative (EC 3.6.3.28)	ISCW006658
Chitinase, putative (EC 3.2.1.14)	ISCW016325
Alternative splicing factor SRp20/9G8, putative	ISCW013289
Ubiquitin-conjugating enzyme, putative (EC 6.3.2.19)	ISCW012877
Ves G 1 allergen, putative (EC 3.1.1.3)	ISCW011596
Transmembrane protein, putative	ISCW011600
**DOWNREGULATED IN *I. SCAPULARIS* BUT UPREGULATED IN *I. RICINUS* CELLS**	
Inositol-1-phosphate synthetase, putative (EC 5.5.1.4)	ISCW018182
Monocarboxylate transporter, putative	ISCW007904
PHD-finger containing protein	ISCW005593
Acetylcholinesterase, putative (EC 3.1.1.1)	ISCW013301
Cytochrome P450, putative (EC 1.14.15.6)	ISCW021866
Cyclophilin type peptidyl-prolyl cis-trans isomerase (EC 5.2.1.8)	ISCW019968
Glyoxylate/hydroxypyruvate reductase, putative (EC 1.1.1.26)	ISCW019963
Synaptotagmin-14, putative (EC 2.7.1.154)	ISCW019969
Secreted protein, putative	ISCW022444
WD-repeat protein, putative (EC 2.7.11.7)	ISCW015562
Nudix hydrolase, putative (EC 3.6.1.22)	ISCW015557
Molecular chaperone, putative	ISCW015579
ATPase, putative	ISCW015572
SMC protein, putative (EC 1.3.1.74) (Fragment)	ISCW015559
Prefoldin, putative	ISCW008190
Multidrug resistance protein, putative (EC 3.6.3.44)	ISCW008199
Amino acid transporter	ISCW002187
Centromere protein B, putative	ISCW017904
Protein NUF1, putative (EC 1.3.1.74)	ISCW007413
U1 small nuclear ribonucleoprotein, putative	ISCW021696
Pygopus, putative	ISCW012547
Beta chain of the tetrameric hemoglobin, putative	ISCW012561
Oviductin, putative (EC 3.4.21.71) (EC 3.4.24.19)	ISCW012546
Flavin-containing monooxygenase, putative (EC 1.14.13.8)	ISCW012562
Synaptotagmin-14, putative (EC 2.7.1.154)	ISCW019969
Chaperonin complex component, TCP-1 eta subunit, putative	ISCW023397

**Figure 1:**
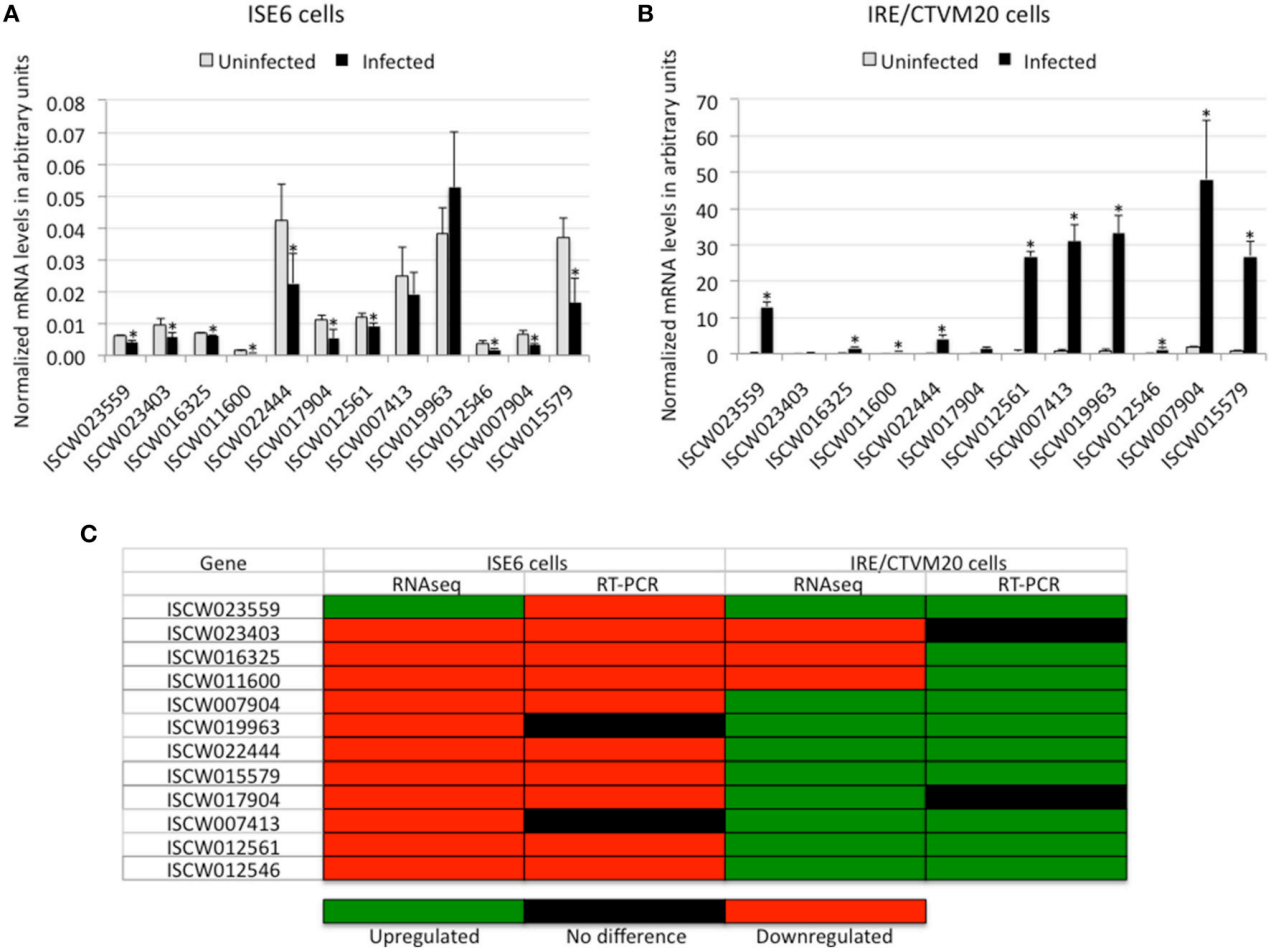
**Validation of RNAseq results**. **(A,B)** The expression of selected differentially expressed genes was characterized by real-time RT-PCR using total RNA extracted from infected and uninfected ISE6 and IRE/CTVM20 tick cells. The mRNA levels were normalized against tick 16S rRNA and cyclophilin using the genNorm method. Normalized *Ct*-values were represented as average + S.D. and compared between infected and uninfected tick cells by Student's *t*-test with unequal variance (^*^P < 0.05; *N* = 5 biological replicates). **(C)** Comparison between RNAseq and real-time RT-PCR results showing a good correlation between both methods.

Twelve genes differentially expressed in both tick cell lines were selected for real-time RT-PCR to validate RNAseq data (Figures 1A,B). The results showed a 75% (9/12) and 67% (8/12) correlation between RNAseq and RT-PCR results for *I. scapularis* ISE6 and *I. ricinus* IRE/CTVM20 cells, respectively (Figure 1C). These results validated the RNAseq data and were similar to those obtained in previous experiments (Ayllón et al., 2015a; Villar et al., 2015a). As previously discussed, the differences observed between the results of the two analyses could be attributed to intrinsic variations in gene expression (Ayllón et al., 2015a).

### Biological process and molecular function of the differentially expressed genes in *I. scapularis* ISE6 and *I. ricinus* IRE/CTVM20 cells

**Figure 2:**
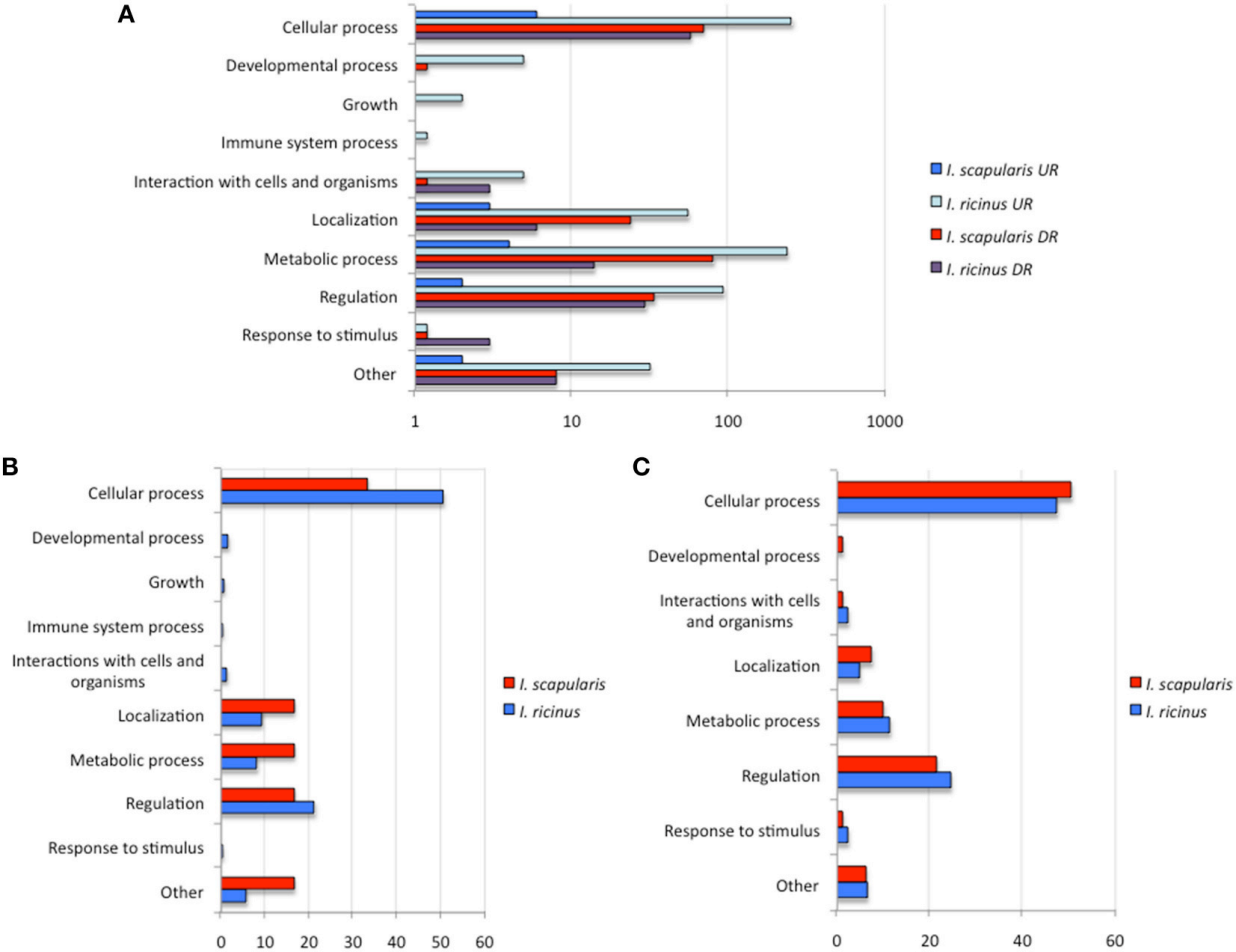
**Biological processes affected by *A. phagocytophilum* infection of *I. ricinus* IRE/CTVM20 and *I. scapularis* ISE6 cells**. **(A)** Differentially expressed genes were functionally annotated and grouped according to the BP of the encoded proteins. The number of genes on each BP is shown. **(B)** Percentage of upregulated genes in each BP. **(C)** Percentage of downregulated genes in each BP. UR, upregulated genes; DR, downregulated genes.

**Figure 3:**
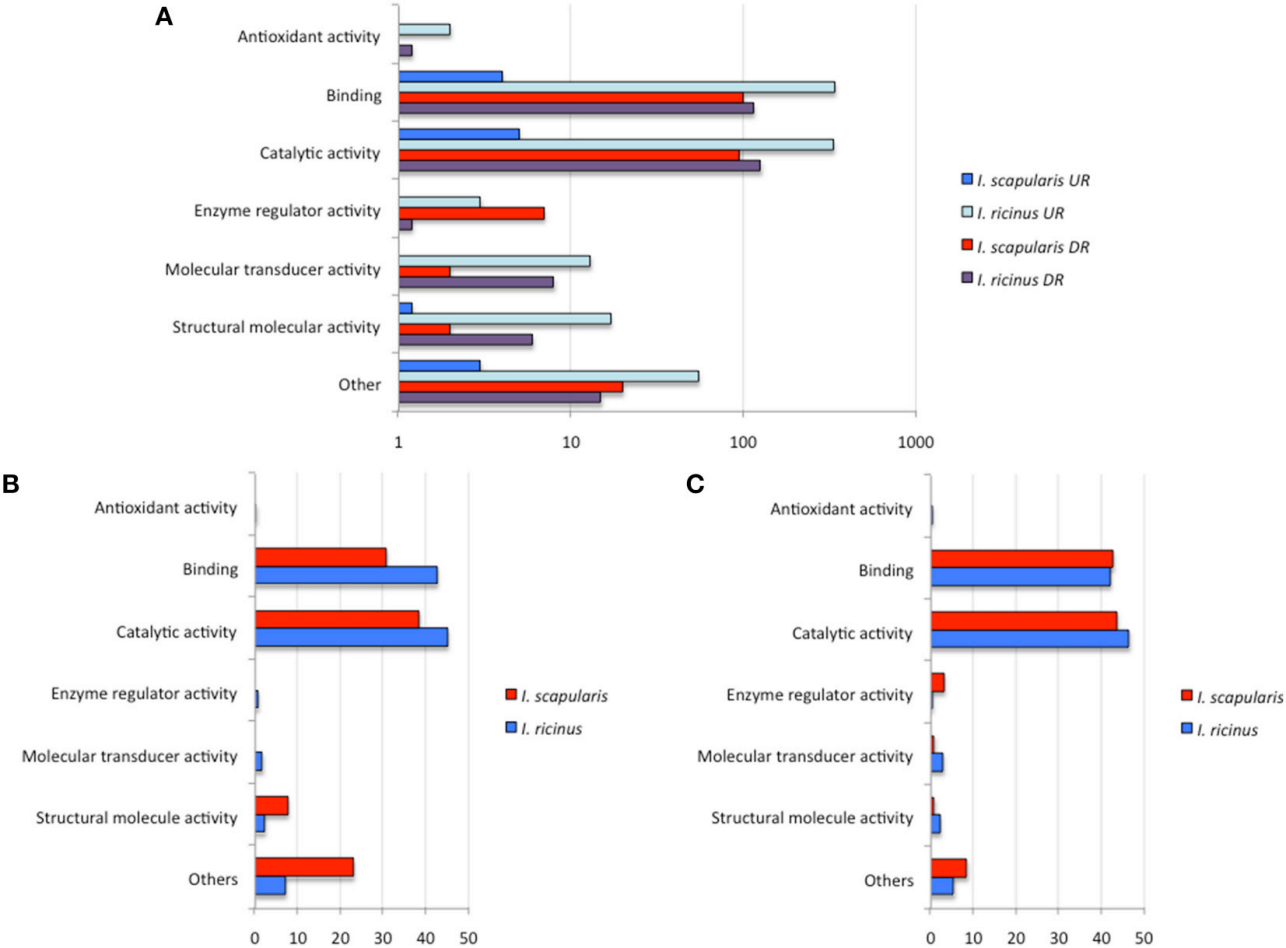
**Molecular function of differentially expressed genes in response to *A. phagocytophilum* infection of *I. ricinus* IRE/CTVM20 and *I. scapularis* ISE6 cells**. **(A)** Differentially expressed genes were functionally annotated and grouped according to the MF of the encoded proteins. The number of genes on each MF is shown. **(B)** Percentage of upregulated genes in each MF. **(C)** Percentage of downregulated genes in each MF. UR, upregulated genes; DR, downregulated genes.

To further characterize differentially expressed genes in response to *A. phagocytophilum* infection, genes were grouped by encoded proteins and annotated by GO to a single BP or MF to avoid redundancy. The analysis of BP showed that the most represented BP in both cell lines corresponded to cellular processes (50 and 33% of the upregulated genes and 48 and 50% of the downregulated genes in *I. ricinus* IRE/CTVM20 and *I. scapularis* ISE6 cells, respectively) and regulation (22 and 18% of the upregulated genes and 25 and 22% of the downregulated genes in *I. ricinus* IRE/CTVM20 and *I. scapularis* ISE6 cells, respectively; Figures 2A–C). The analysis of MF showed that the most represented MF in both cell lines corresponded to binding (43 and 30% of upregulated genes and 43 and 44% of the downregulated genes in *I. ricinus* IRE/CTVM20 and *I. scapularis* ISE6 cells, respectively) and catalytic activity (45 and 38% of upregulated genes and 47 and 43% of the downregulated genes in *I. ricinus* IRE/CTVM20 and *I. scapularis* ISE6 cells, respectively; Figures 3A–C). These results suggested that the most affected BP and MF in response to *A. phagocytophilum* infection were similar in *I. ricinus* IRE/CTVM20 and *I. scapularis* ISE6 cells.

**Figure 4:**
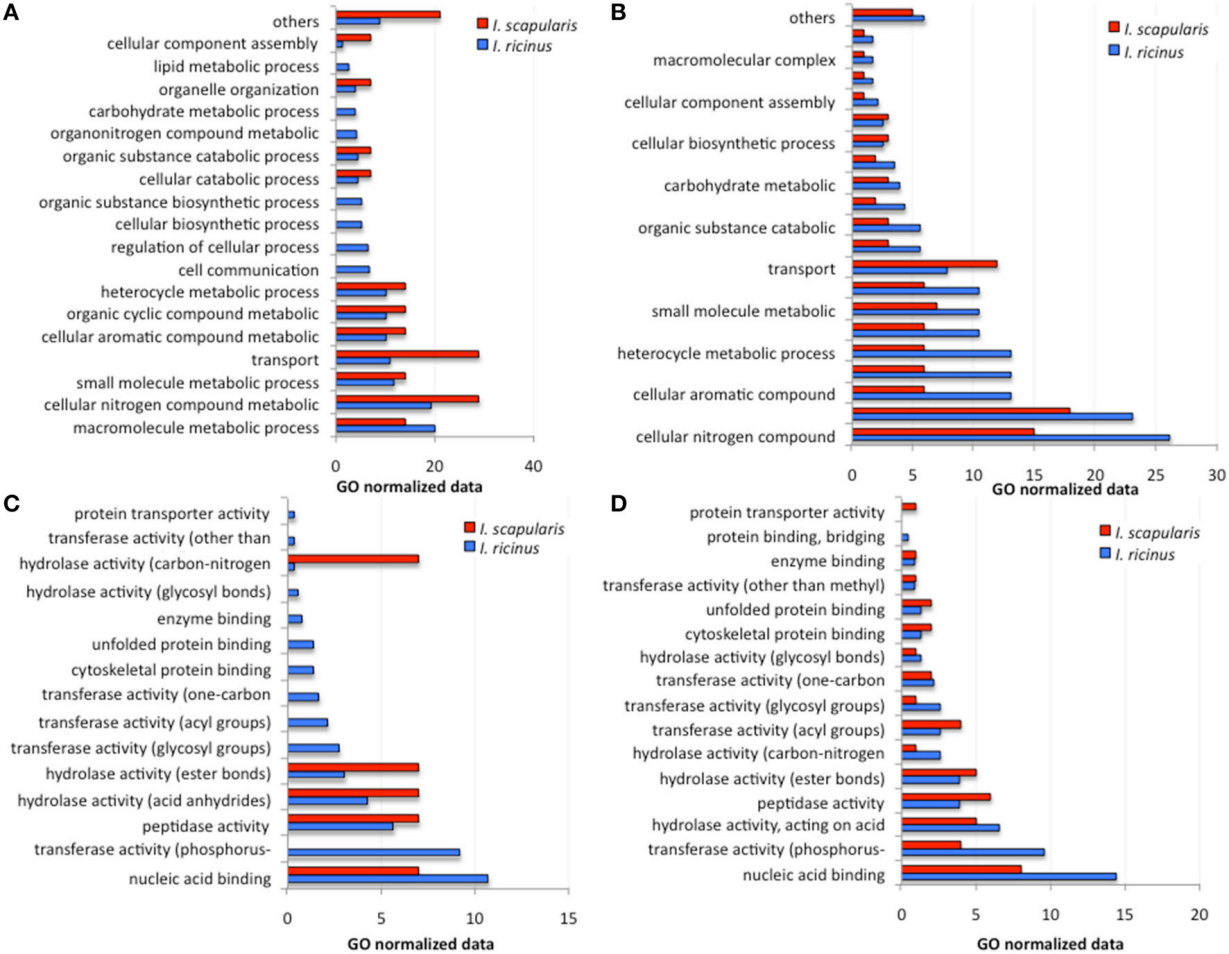
**GO normalized data for differentially expressed genes in response to *A. phagocytophilum* infection of *I. ricinus* IRE/CTVM20 and *I. scapularis* ISE6 cells**. **(A)** BP for upregulated genes. **(B)** BP for downregulated genes. **(C)** MF for upregulated genes. **(D)** MF for downregulated genes. For all BP and MF categories in both upregulated and downregulated genes, values were compared between tick cell lines by Chi^2^ test and resulted in significant differences at *p* < 0.01 for all except for MF of downregulated genes in which differences were not significant between both tick cell lines.

However, when the analysis was conducted with GO normalized data (Figures 4A–D) significant differences between *I. ricinus* IRE/CTVM20 and *I. scapularis* ISE6 cells were observed for both upregulated and downregulated genes in BP (Figures 4A,B) and MF (Figures 4C,D) categories. In general, cell metabolic processes were the most affected BP in both cell lines for upregulated (Figure 4A) and downregulated (Figure 4B) genes. However, significant differences were observed between *I. ricinus* IRE/CTVM20 and *I. scapularis* ISE6 cells for all BP (*p* < 0.01; Figures 4A,B). For MF categories, upregulated but not downregulated genes were significantly different between *I. ricinus* IRE/CTVM20 and *I. scapularis* ISE6 cells (*p* < 0.01; Figures 4C,D).

**Figure 5:**
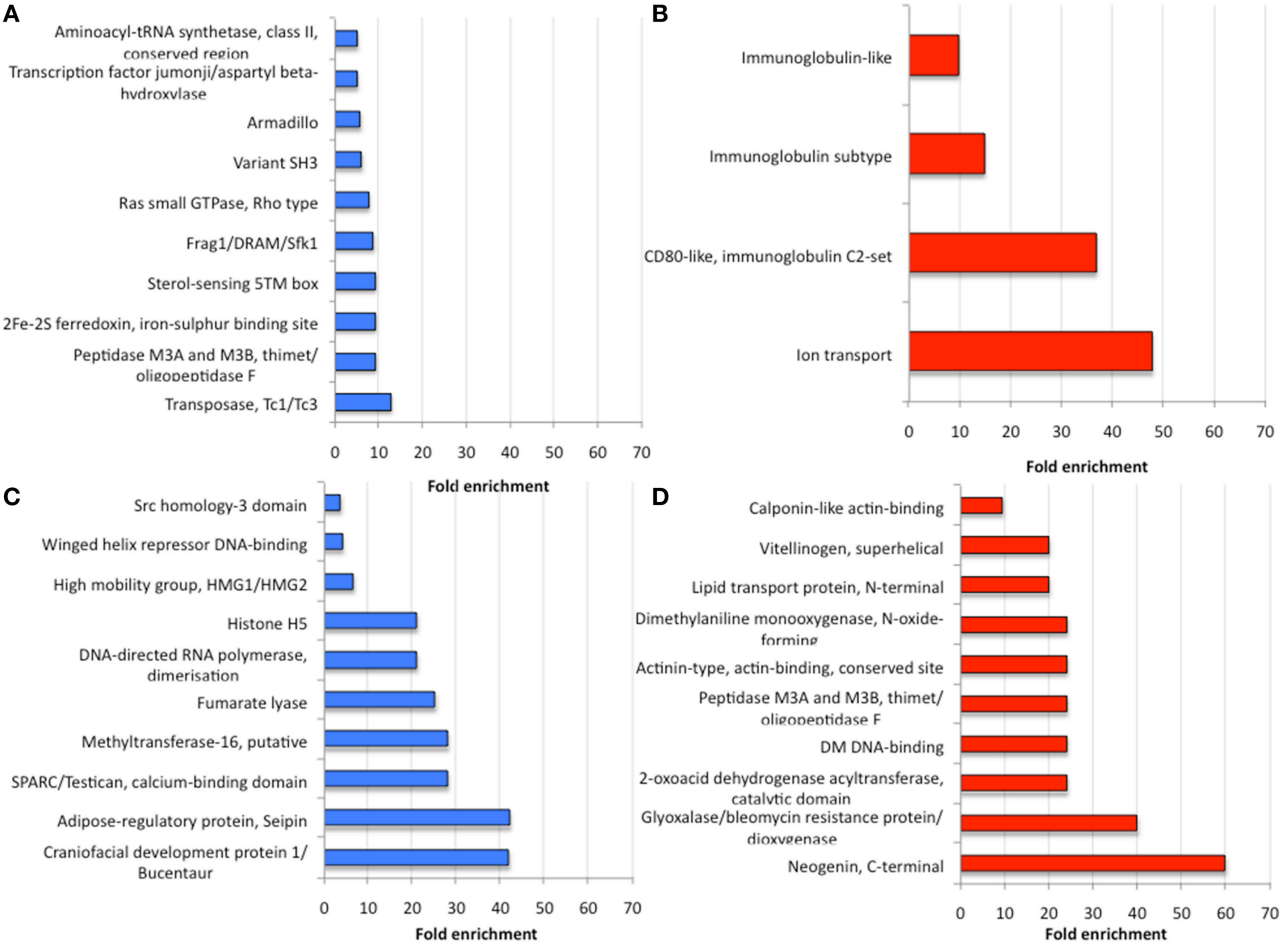
**Analysis of most differentially expressed genes in response to *A. phagocytophilum* infection of *I. ricinus* IRE/CTVM20 and *I. scapularis* ISE6 cells**. **(A)** Proteins encoded by the 10 most upregulated genes in *I. ricinus* IRE/CTVM20 cells. **(B)** Proteins encoded by the 4 most upregulated genes in *I. scapularis* ISE6 cells. **(C)** Proteins encoded by the 10 most downregulated genes in *I. ricinus* IRE/CTVM20 cells. **(D)** Proteins encoded by the 10 most downregulated genes in *I. scapularis* ISE6 cells. The InterPro motifs obtained using DAVID were used to evaluate the fold enrichment of differentially expressed genes in the corresponding tick cell line (*p* ≤ 0.05).

Focusing on the most upregulated and downregulated genes in response to *A. phagocytophilum* infection, similarities and differences were found between *I. ricinus* IRE/CTVM20 and *I. scapularis* ISE6 cells. For example, the gene coding for Voltage-gated ion channel was among the upregulated genes in both tick cell lines (number of sequences = 2; FE>5; *p* = 7.8E-03; Table 1). It has been reported that changes in intracellular ion concentrations is required for the activation and function of apoptosis but also that ionic homoeostasis is essential in response to changes in extracellular environment (Hoffmann et al., 2009; Bortner and Cidlowski, 2014). Therefore, upregulation of the Voltage-gated ion channel was probably a of tick cell response to infection. In other organisms, Voltage-gated calcium channels play an important role in pathogen infection (Gupta et al., 2009; Lavanya et al., 2013). In ticks, Voltage-gated calcium channel levels could be manipulated by *A. phagocytophilum* to regulate key intracellular second messengers such as calcium to evade protective immune responses or protein exocytosis favoring pathogen transmission (Maritz-Olivier et al., 2005). However, most of the highly upregulated (Figures 5A,B) and downregulated (Figures 5C,D) genes showed a significantly different FE (*p* ≤ 0.05) between *I. ricinus* IRE/CTVM20 and *I. scapularis* ISE6 cells.

### Tissue-specific signatures in transcriptional response to *A. phagocytophilum* infection in *I. scapularis* ISE6 and *I. ricinus* IRE/CTVM20 cells

These results showed functionally relevant differences in the response of *I. ricinus* IRE/CTVM20 and *I. scapularis* ISE6 cells to *A. phagocytophilum* infection. Tick cell lines are a valuable tool for the study of tick biology and tick-pathogen interactions (Bell-Sakyi et al., 2007; Villar et al., 2015a,b; Weisheit et al., 2015). However, differences have been reported between *in vivo* and *in vitro* experiments, probably associated with differences in the cell populations represented in different cell lines (Munderloh et al., 1994; Alberdi et al., 2015; Villar et al., 2015a; Weisheit et al., 2015).

The *I. scapularis* ISE6 cells, which constitute a model for hemocytes involved in pathogen infection and immune response (Munderloh et al., 1994), produce hemolymph effector proteins associated with tick hemocytes and involved in *A. phagocytophilum* infection such as P11 and the related protein B7P9I7, Defensins, Complement-like molecules, and Antimicrobial peptides (Villar et al., 2015a). As expected, the results of the present study correlated with those reported previously using integrated metabolomics, transcriptomics, and proteomics data (Villar et al., 2015a) in which cellular processes such as immune response, protein processing and glucose metabolism were affected by *A. phagocytophilum* infection of *I. scapularis* ISE6 cells (Figures 5B,D). Furthermore, among the most abundant transcripts differentially expressed in infected *I. scapularis* ISE6 cells were those encoding Vitellinogen, Lipid transport protein, Monooxygenase, and Immunoglobulin-like proteins that have been previously described in tick hemolymph (Gudderra et al., 2002; Kadota et al., 2002; Figures 5B,D). Therefore, the transcriptional response of ISE6 cells to *A. phagocytophilum* infection may be similar to that observed in tick hemocytes, thus supporting a tissue-specific signature for ISE6 cells (Villar et al., 2015a).

**Figure 6:**
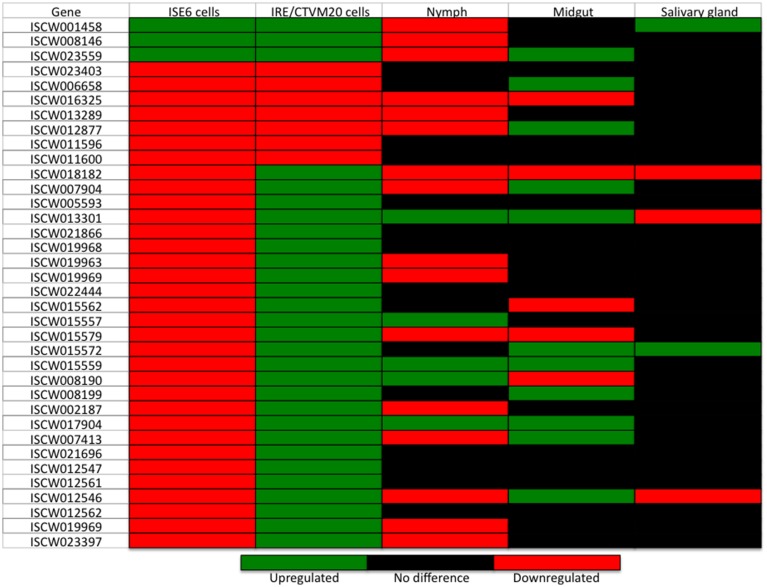
**Transcriptional profile of genes differentially expressed in both *A. phagocytophilum*-infected *I. ricinus* IRE/CTVM20 and *I. scapularis* ISE6 cells and in comparison with infected tick samples**. Differential expression (*p* ≤ 0.05; *q* ≤ 0.05) is shown for *I. scapularis* ISE6 cells, *I. ricinus* IRE/CTVM20 cells, and *I. scapularis* nymphs and adult midguts and salivary glands. Data for tick samples was obtained from Ayllón et al. (2015a).

In order to define a tissue-specific signature for the transcriptional response of *I. ricinus* IRE/CTVM20 cells to *A. phagocytophilum* infection, a preliminary analysis was undertaken by comparing the expression of genes differentially expressed in both cell lines with that reported previously in *I. scapularis* tick samples (Ayllón et al., 2015a; Figure 6). The results demonstrated that in infected *I. ricinus* IRE/CTVM20 cells, 22, 28, and 6% of the genes had a similar response to that in infected tick nymphs, midguts and salivary glands, respectively. In infected *I. scapularis* ISE6 cells, 36, 17, and 11% of the genes had a similar response to that in infected tick nymphs, midguts and salivary glands, respectively. Furthermore, the proteins encoded by highly upregulated and downregulated genes in response to *A. phagocytophilum* infection in *I. ricinus* IRE/CTVM20 cells had MF associated with catalytic activity, binding, and structural molecules (Figures 5A,C), which were also identified in tick midguts in response to infection (Ayllón et al., 2015a). These results suggested that while *I. ricinus* IRE/CTVM20 cells responded to infection more like tick midguts, *I. scapularis* ISE6 cells response to infection appeared more closely related to that in nymphs, the only sample in which hemolymph containing tick hemocytes was present.

### The inhibition of tick cell apoptosis by *A. phagocytophilum* correlates with tissue-specific signatures in transcriptional response to infection in *I. scapularis* ISE6 and *I. ricinus* IRE/CTVM20 cells

The inhibition of cell apoptosis by *A. phagocytophilum* appears to be a key adaptation mechanism to facilitate infection of both vertebrate and tick cells and was used to investigate further the tissue-specific response of tick cell lines to pathogen infection (Ayllón et al., 2013, 2015a; Severo et al., 2015; Villar et al., 2015a; de la Fuente et al., 2016).

**Figure 7:**
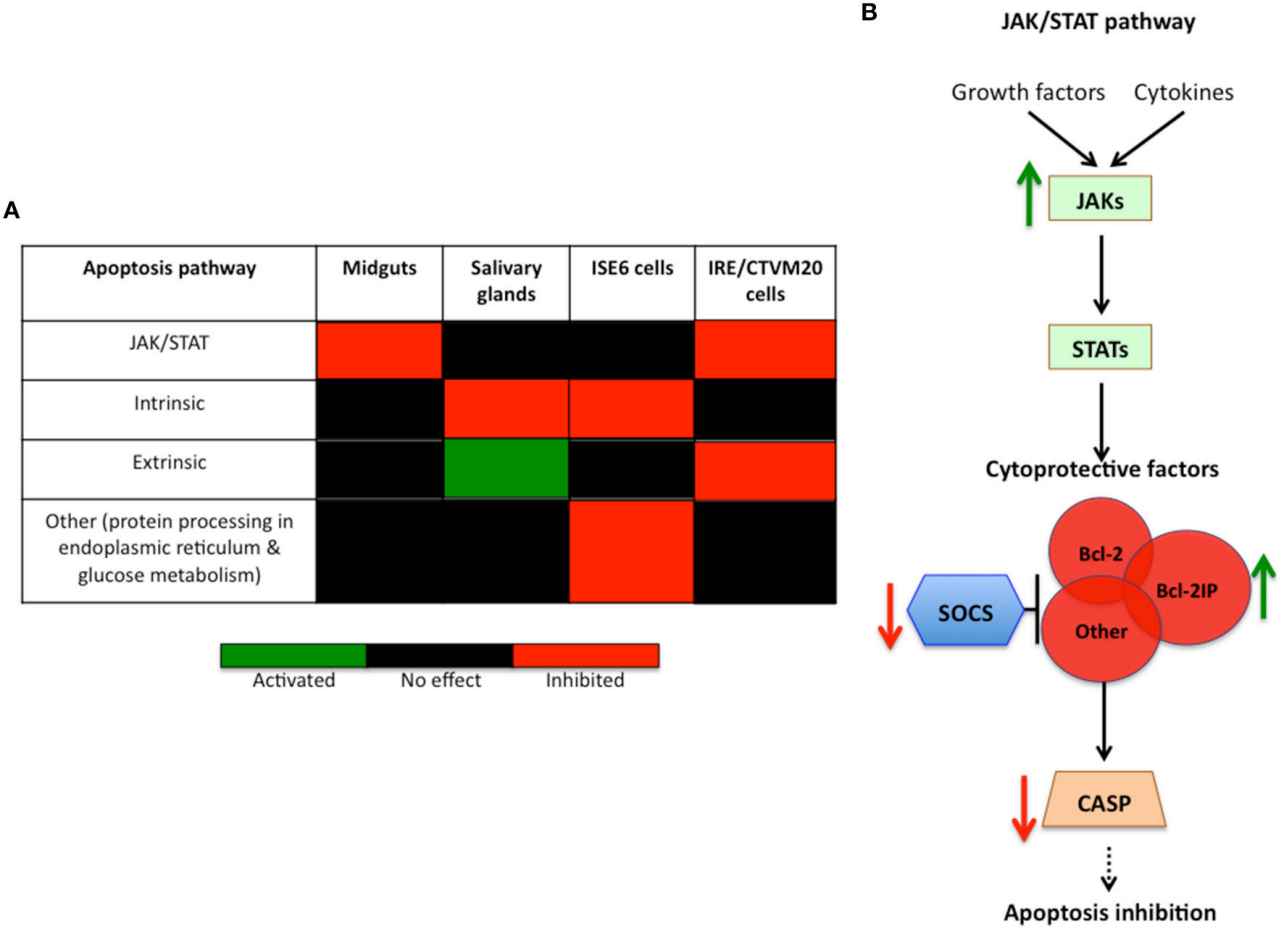
**Tissue-specific signatures in the inhibition of tick cell apoptosis by *A. phagocytophilum*. (A)** Tissue-specific response in the inhibition of tick cell apoptosis by *A. phagocytophilum*. The annotation of JAK/STAT pathway genes and the effect of *A. phagocytophilum* infection on tick tissue response were obtained from previous reports (Ayllón et al., 2015a; Villar et al., 2015a). **(B)** Schematic representation of the effect of *A. phagocytophilum* on the inhibition of the JAK/STAT pathway to establish infection in *I. ricinus* IRE/CTVM20 cells. In infected *I. ricinus* IRE/CTVM20 cells, transcriptomics results reported here showed upregulation of JAKs resulting in activation of the JAK/STAT pathway. Additionally, downregulation of SOCS enhances the effect of induced cytoprotective factors such as Bcl-2IP, which in turn reduce CASP expression to inhibit apoptosis.

As previously reported in *I. scapularis* ISE6 tick cells (Ayllón et al., 2013; Alberdi et al., 2015; Villar et al., 2015a), *A. phagocytophilum* infection inhibits tick cell apoptosis through different mechanisms including downregulation of *porin* expression resulting in lower Cytochrome c protein levels as a mechanism to inhibit the intrinsic apoptosis pathway and facilitate infection (Figure 7A). Although not identified previously in *I. scapularis* nymphs or adult female midguts and salivary glands in response to *A. phagocytophilum* infection (Ayllón et al., 2013), downregulation of neogenin (ISCW023402) in ISE6 tick cells (Figure 5D and Supplementary Table 2) suggested a new mechanism by which bacterial infection inhibits apoptosis to facilitate infection (Matsunaga et al., 2004). However, in *I. ricinus* IRE/CTVM20 cells the inhibition of apoptosis appeared to be regulated by lower Caspase (CASP) protein levels in infected tick cells (Alberdi et al., 2015).

To further characterize apoptosis inhibition by *A. phagocytophilum* in *I. ricinus* IRE/CTVM20 cells, the transcriptional profile of apoptosis pathway genes was obtained from RNAseq data. The results showed downregulation of *suppressor of cytokine signaling* (SOCS; ISCW019429) and upregulation of *Janus kinase* (JAK; ISCW016145) involved in activation of the Janus kinase/signal transducers and activators of transcription (JAK/STAT) pathway, upregulation of *fatty acid synthase* (FAS; ISCW009053) implicated in the extrinsic apoptosis pathway, and upregulation of *cytochrome c* (CYTC; ISCW008740) and *bcl-2 interacting protein* (Bcl-2IP; ISCW008098) of the intrinsic apoptosis pathway (Supplementary Table 5).

These results did not support a role for the intrinsic pathway in the inhibition of cell apoptosis by *A. phagocytophilum* infection of *I. ricinus* IRE/CTVM20 cells, a mechanism previously described in *I. scapularis* tick salivary glands and ISE6 cells (Ayllón et al., 2013, 2015a; Alberdi et al., 2015; Figure 7A). In contrast, the results in *I. ricinus* IRE/CTVM20 cells were similar to those obtained in *I. scapularis* midguts after infection with *A. phagocytophilum* (Ayllón et al., 2015a) which together with previous results in tick cells (Alberdi et al., 2015) suggested a role for the JAK/STAT pathway in the inhibition of apoptosis in *I. ricinus* IRE/CTVM20 infected cells (Negoro et al., 2000; Croker et al., 2008; Figure 7A). In *I. ricinus* IRE/CTVM20 cells, *A. phagocytophilum* infection resulted in the upregulation of JAK probably inducing the activation of the JAK/STAT pathway. Additionally, downregulation of SOCS may enhanced the effect of induced cytoprotective factors such as Bcl-2IP, which in turn could reduced CASP expression to inhibit apoptosis (Figure 7B). Additionally, the upregulation of *fatty acid synthase* suggested a possible effect of *A. phagocytophilum* infection on the inhibition of the extrinsic apoptosis pathway (Figure 7A), but additional experiments are required to support this result.

## Conclusions

The results obtained in the present study provided support for our hypothesis and suggested the presence of tissue-specific signatures in tick cell line response to *A. phagocytophilum* infection. The transcriptional response to infection of *I. scapularis* ISE6 cells resembled that of tick hemocytes while the response in *I. ricinus* IRE/CTVM20 was more closely related to that reported previously in infected tick midguts. Tick infection by *A. phagocytophilum* results in the inhibition of cell apoptosis to facilitate pathogen infection and multiplication and was therefore used to investigate further the tissue-specific response of tick cell lines to pathogen infection. The results supported a role for the intrinsic pathway in the inhibition of cell apoptosis by *A. phagocytophilum* infection of *I. scapularis* ISE6 cells. In contrast, the results in *I. ricinus* IRE/CTVM20 cells were similar to those obtained in tick midguts and suggested a role for the JAK/STAT pathway in the inhibition of apoptosis in tick cells infected with *A. phagocytophilum*. However, tick cell lines were derived from embryonated eggs and may contain various cell populations with different morphology and behavior that could affect transcriptional response to infection (Munderloh et al., 1994; Bell-Sakyi et al., 2007). Furthermore, studies comparing infected *I. scapularis* and *I. ricinus* ticks are needed to fully address possible differences between tick species in response to *A. phagocytophilum* infection. Nevertheless, the results reported here support the use of tick cell lines as models to study tissue-specific response to pathogen infection.

## Author contributions

JF, NJ, AF conceived the experiment. PA, KM, RR, CC, NA, MV conducted the experiments. JF, KM, RR, NJ, AF wrote the paper.

## Funding

This research was supported by grant BFU2011-23896 from Ministerio de Economía y Competitividad (MINECO), Spain and the European Union FP7 ANTIGONE project number 278976. NA was funded by MINECO, Spain. LMH was supported by a fellowship from the University of Castilla La Mancha (UCLM), Spain. MV was supported by the Research Plan of UCLM, Spain.

### Conflict of interest statement

The authors declare that the research was conducted in the absence of any commercial or financial relationships that could be construed as a potential conflict of interest.

## Supplementary material

Supplementary Table 1 **Sequences of oligonucleotide primers used for real-time RT-PCR**. pdf.

Supplementary Table 2 **Differentially expressed genes in *I. scapularis* ISE6 cells**. xlsx.

Supplementary Table 3 **RNAseq statistics for *I. ricinus* IRE/CTVM20 cells**. pdf.

Supplementary Table 4 **Transcriptomics results for *I. ricinus* IRECTVM20 cells**. xlsx.

Supplementary Table 5 **Differentially expressed genes in *I. ricinus* IRECTVM20 cells**. xlsx.
