# Supplementary material for: Tissue-Specific Signatures in the Transcriptional Response to Anaplasma phagocytophilum Infection of Ixodes scapularis and Ixodes ricinus Tick Cell Lines
Source: Front Cell Infect Microbiol. 2016 Feb 10;6:20. doi: 10.3389/fcimb.2016.00020 (PMC4748044; doi:10.3389/fcimb.2016.00020)
Supplement: Supplementary Table 1 — Sequences of oligonucleotide primers used for real-time RT-PCR. pdf. [file Table1.PDF]

Supplementary Table 1. Sequences of oligonucleotide primers used for real-time RT-PCR.

Gene	Genbank accession No.	Forward and reverse primers (5'-3')
voltage-gated ion channel	ISCW023559	CAGTACGCCGATCACCACAT<br>TGAACATGCCCGGAAGGAAA
neural cell adhesion molecule L1	ISCW023403	CTACGGTGAGGGGTCAGAGA<br>GTTCGGACGAGGGTTTCCAT
chitinase	ISCW016325	ATAACCTGGATGGCGTGGAC<br>GTCCAACCGCTCTTGAGGAA
transmembrane protein	ISCW011600	ACGTTGTCAACCCCAACACT<br>CGGGTGCCACAACTTGAATG
secreted protein	ISCW022444	CTACATCCGGGAATCGTCCG<br>CTCACCACCACTGGAAGCAT
centromere protein B	ISCW017904	GGCCACCTCCCTGAACTATG<br>CGCGTGCAAGTACGAGAAAG
beta chain of the tetrameric hemoglobin	ISCW012561	CTCCATCATCGACACCCTCG<br>GCTCCCGATATGGTGGGATG
protein NUF1	ISCW007413	ATGCCAGAGAGGTTGCTGTC<br>CTTCTCCCTCAGCAGTGTCG
glyoxylate/hydroxypyruvate reductase	ISCW019963	ATCACGGCAGGTGACAAACT<br>GGGTGTTCGTCACCGGAATA
oviductin	ISCW012546	AAAGCTGACCGATTCCAGCA<br>TGGGAGATCCCGTCGTAGTT
monocarboxylate transporter	ISCW007904	CTTGGTGGACATCCTCGGTC<br>GTCGTACAGCCAACCTGTCA
molecular chaperone	ISCW015579	ATGGCGACCTCTTCGGTTG<br>CGATCTGTTGTTTGGGCGTG
ribosomal protein S4	DQ066214	GGTGAAGAAGATTGTCAAGCAGAG<br>TGAAGCCAGCAGGGTAGTTTG
