# Supplementary material for: Tissue-Specific Signatures in the Transcriptional Response to Anaplasma phagocytophilum Infection of Ixodes scapularis and Ixodes ricinus Tick Cell Lines
Source: Front Cell Infect Microbiol. 2016 Feb 10;6:20. doi: 10.3389/fcimb.2016.00020 (PMC4748044; doi:10.3389/fcimb.2016.00020)
Supplement: Supplementary Table 3 — RNAseq statistics for I. ricinus IRE/CTVM20 cells. pdf. [file Table3.PDF]

Supplementary Table 3. RNAseq statistics for *I. ricinus* IRE/CTVM20 cells.

**Sample**	**Uninfected replicate 1/replicate 2**	**Infected replicate 1/replicate 2**
TOTAL NO. READS	14238098/15139730	13405605/15337239
ALIGNED READS	9409441/9946872	8874022/10074225
PF_READS	14238098/15139730	13405605/15337239
PF_ALIGNED READS	14238098/15139730	13405605/15337239
PF_HQ_ALIGNED READS	5582184/6540394	5599833/6501436
MEAN READ LENGTH	119/119	119/119
PF_HQ_ERROR_RATE	0.759136/0.761209	0.761739/0.760755
PF_INDEL_RATE	0.000572/0.000578	0.000604/0.000614

Abbreviations: PF, pass-Illumina filter; HQ, high quality; PF_HQ_ERROR_RATE: Percentage of *I. scapularis* sequence bases that mismatch the *I. scapularis* reference genome sequence in PF HQ aligned reads; PF_INDEL_RATE: Number of insertion and deletion events per 100 PF aligned bases. It uses the number of events as the numerator, not the number of inserted or deleted bases. The following command was used for analysis: samtools view –F 0x4 sortedbamfile.bam | cut –f 1 | sort | uniq | wc –l (http://www.htslib.org). Picard metrics definitions (http://picard.sourceforge.net/picard-metric-definitions.shtml) were used.
